# Dose-volume relationships for moderate or severe neck muscle atrophy after intensity-modulated radiotherapy in patients with nasopharyngeal carcinoma

**DOI:** 10.1038/srep18415

**Published:** 2015-12-18

**Authors:** Lu-Lu Zhang, Xiao-Ju Wang, Guan-Qun Zhou, Ling-Long Tang, Ai-Hua Lin, Jun Ma, Ying Sun

**Affiliations:** 1 Department of Radiation Oncology, Sun Yat-sen University Cancer Center, State Key Laboratory of Oncology in South China, Collaborative Innovation Center for Cancer Medicine, 651 Dongfeng Road East, Guangzhou 510060, People’s Republic of China; 2 Department of Medical Statistics and Epidemiology, School of Public Health, Sun Yat-sen University, Guangzhou, People’s Republic of China

## Abstract

This study aimed to identify the dosimetric parameters and radiation dose tolerances associated with moderate or severe sternocleidomastoid muscle (SCM) atrophy after intensity-modulated radiotherapy (IMRT) in nasopharyngeal carcinoma (NPC). We retrospectively analysed 138 patients treated with IMRT between 2011 and 2012 for whom IMRT treatment plans and pretreatment and 3-year post-IMRT MRI scans were available. The association between mean dose (Dmean), maximum dose (Dmax), VX (% SCM volume that received more than X Gy), DX (dose to X% of the SCM volume) at X values of 20, 25, 30, 35, 40, 45, 50, 55, 60, 65, 70, 75, 80 and SCM atrophy at 3 years after IMRT were analyzed. All dosimetric parameters, except V40, V50 and V80, were significantly associated with moderate or severe SCM atrophy. Multivariate analysis showed that V65 was an independent predictor of moderate or severe SCM atrophy (*P* < 0.001). Receiver operating characteristic (ROC) curve indicated a V65 of 21.47% (area under ROC curves, 0.732; *P* < 0.001) was the tolerated dose for moderate or severe SCM atrophy. We suggest a limit of 21.47% for V65 to optimize NPC treatment planning, whilst minimizing the risk of moderate or severe SCM atrophy.

Nasopharyngeal carcinoma (NPC) is a head and neck malignancy with unique epidemiological features. Worldwide, southern China and southeast Asia have the highest incidence of NPC. Here the reported annual incidence is 20–30 cases per 10^5^ individuals^1^,^2^,^3^. As a consequence both of the surgical approach to the nasopharynx being difficult, and the fact the NPC is highly sensitive to ionizing radiation, radical radiotherapy is the usual primary treatment for all stages of the disease which are locally or regionally confined^4^. With the superior local control rate achieved in patients treated with intensity-modulated radiation therapy (IMRT)^5^,^6^, late complications and long-term quality of life after radiotherapy are now receiving more attention.

In clinical practice when designing IMRT treatment plans, dose constraints are required to limit the radiation dose received by particular organs at risk (OARs), and this measure has been proven to effectively protect many OARs such as the optic nerve, optic chiasm, brain stem and spinal cord^7^,^8^. IMRT can reduce the radiation dose to selected dose-limiting organs; however, at the same time, this may increase the dose of radiation received by other undefined tissues^9^. Irradiation of the positive neck nodes and entire cervical lymph drainage area is the traditional practice for treating NPC^10^. However, an OAR dose limit for the neck muscles has not yet been established and is probably the main factor leading to neck muscle atrophy.

The sternocleidomastoid muscle (SCM), which begins at the medial and manubrium end of the clavicle and ends in the mastoid process of the temporal bone, is one of the major superficial cervical flexor muscles and prime movers of the neck^11^ and plays an important role in turning the head towards the side opposite the contracting muscles. Thus, the impact of SCM injury on the normal motion of the neck can be enormous. However, IMRT-induced SCM atrophy, which may contribute to the development of weakness and further impair quality of life, has received little investigation. The precise mechanism regarding radiotherapy-induced neck muscle atrophy remains unknown, but it is believed that the incidence and severity of neck muscle atrophy is associated with volume and dose of neck muscle irradiated^12^.

Hence, we conducted this retrospective study to investigate the incidence of SCM atrophy in patients with NPC at three years after IMRT and also investigate the dose-response relationships for moderate or severe SCM atrophy using a dose-volume-outcome analysis. The purpose of this study was to offer guidelines for optimizing IMRT treatment plans in order to reduce the incidence of neck muscle atrophy and weakness in patients with NPC.

## Materials and Methods

### Patient selection

Between January 2011 and January 2012, 549 newly diagnosed patients with NPC with no distant metastases were treated using IMRT at our centre. A total of 355 patients were excluded due to the IMRT treatment plan documents being unavailable. Forty-three patients were excluded because they had not had MRI scans pre-treatment and/or 3 years post-IMRT. Of the remaining 151 patients, 6 were excluded due to them having diabetes mellitus, 2 were excluded due to them having connective tissue disorders; and 5 were excluded due to them having had previous neck surgery. From the original 549 patients, 138 were included in this study. This retrospective study was approved by the Institutional Review Board of Sun Yat-sen University Cancer Center, and was conducted in accordance with Good Clinical Practice guideline. Informed consent was obtained from all patients.

Pretreatment baseline evaluation included a complete medical history, physical examination, haematology and biochemistry profiles, MRI of the neck and nasopharynx, chest radiography, abdominal sonography, and bone scan emission computed tomography (SPECT). All patients were staged as part of their routine treatment according to the 7th edition of the AJCC staging system^13^. The clinical features of the patients are summarised in Table 1.

### Treatment methods

All patients underwent radical IMRT treatment. The target volumes were delineated according to our institutional protocol, which is in agreement with the International Commission on Radiation Units and Measurements Reports 50 and 62^14^. In summary, the prescribed dose was limited to 68–70 Gy in 30–33 fractions to the planning target volume (PTV) of the gross tumour volume of the primary (GTV-P), 60–68 Gy to the PTV of the nodal gross tumour volume (GTV-N), 60 Gy to the PTV of (CTV)-1 (high-risk region) and 54 Gy to the PTV of CTV-2 (low risk region and neck nodal regions). IMRT was delivered at one fraction daily over 5 days per week. Induction or adjuvant chemotherapy consisted of cisplatin with 5-fluorouracil or cisplatin with taxanes every three weeks for 1–4 cycles. Concurrent chemotherapy consisted of cisplatin every three weeks or cisplatin weekly. Of the 122/138 (88.41%) patients with stage III/IV NPC, 118/122 (96.72%) received chemotherapy.

### Data collection

#### Muscle volume measurement

All patients underwent MRI using a 1.5-Tesla system (Signa CV/i; General Electric Healthcare, Chalfont St. Giles, United Kingdom). The area from the suprasellar cistern to the inferior margin at the sternal end of the clavicle was examined with a head and neck combined coil, as previously described^15^. Measurement of the muscle volumes of the left and right sides of the SCM was performed separately by volume rendering. MRI scanning images obtained at pretreatment and 3-years post-IMRT for each study subject were conveyed to Advantage Workstation 4.4 (General Electric Healthcare). The outline of the SCM was traced on each T2-weighted axial image, beginning at the manubrium and collarbone and ending at the mastoid, and then the bilateral SCM muscle volumes were calculated by volume rendering. A radiologist and clinician trained to use the software to delineate the SCM contoured the bilateral muscles independently, any disagreements about the SCM delineation were resolved by consensus. Then the average of the measurements was taken as the final muscle volume.

#### Grading of muscle atrophy

The following formula was used to calculate the extent of SCM atrophy: SCM atrophy ratio (%) = [(SCM volume at pre-treatment - SCM volume at 3-years post-IMRT)/SCM volume at pre-treatment] × 100. SCM atrophy was graded according to a 3-point visual grading system as follows: Grade 0, no atrophy; Grade 1, minimal to mild atrophy, 0 < SCM atrophy ratio ≤40%; Grade 2, moderate atrophy, 40% < SCM atrophy ratio ≤70%; Grade 3, severe atrophy, SCM atrophy ratio >70%. This system is similar to that proposed by Hyun Kyong Lim *et al.*^16^. Grade 2 and 3 were defined as the endpoint of the study.

#### Grading of muscle weakness

Neck muscle weakness was assessed at follow-up appointments, approximately 3 years post IMRT. Muscle weakness was graded using the Common Terminology Criteria for Adverse Events (CTCAE) V3.0: grade 0, no weakness; grade 1, asymptomatic, but weakness on physical exam; grade 2, symptomatic weakness interfering with function, but not interfering with activities of daily living; grade 3, symptomatic weakness interfering with activities of daily living; grade 4, life-threatening or disabling weakness; grade 5, death due to muscle weakness.

#### DVH factors for muscles

The following parameters were collected from the dose-volume histograms (DVH) for the bilateral SCM muscles for each patient: mean dose (Dmean), maximum dose (Dmax), VX (the percentage of the SCM volume that received more than X Gy) and DX (the dose to X% of the SCM volume) at X values of 20, 25, 30, 35, 40, 45, 50, 55, 60, 65, 70, 75, 80.

### Follow-up and statistical analysis

Patients were examined at least every 3 months in the first 2 years, and thereafter, patients returned for follow-up every six months (or until death). A complete physical examination, chest radiography and abdominal sonography were performed as routine follow-up, and neck and nasopharyngeal MRI was performed every 6 to 12 months.

All data analysis was performed using SPSS version 13.0 (SPSS, Chicago, IL, USA). The endpoint of this research was the occurrence of moderate or severe (grade 2–3) SCM atrophy. To identify significant dosimetric parameters for moderate or severe SCM atrophy, the dosimetric parameters Dmean, Dmax, D20, D25, D30, D35, D40, D45, D50, D55, D60, D65, D70, D75, D80, V20, V25, V30, V35, V40, V45, V50, V55, V60, V65, V70, V75, V80 were compared in individuals with grade 0–1 SCM atrophy to those in individuals with grade 2–3 SCM atrophy using univariate analysis and the Wilcoxon rank-sum test. Binary logistic regression analysis was used for multivariate analyses to test whether the significant dosimetric parameters identified in univariate analysis were independent risk factors for moderate or severe SCM atrophy. Then, ROC (Receiver operating characteristic) curves were used to assess the dose tolerances for the independent significant factors with respect to moderate or severe SCM atrophy. The relationship between neck muscle atrophy and neck muscle weakness was analyzed using spearman rank correlation analysis. Two-sided *P* values ≤0.05 were considered statistically significant.

## Results

### Patient characteristics and incidence of SCM atrophy

The male/female ratio of the 138 patients was 2.83:1 (102 males; 36 females), and the median age was 42 years-old (range, 14 to 69 years-old). In total, 1.4% (2/138), 10.1% (14/138), 48.6% (67/138), and 39.9% (55/138) of the patients were classified as having Stage I, II, III, and IV disease, respectively. According to the World Health Organization (WHO) classification, 137 of the 138 (99.28%) patients had type II NPC and 1/138 (0.72%) had type I NPC. The clinical characteristics and treatment parameters for the 138 patients are summarised in Table 1.

The grades of muscle atrophy at 3 years post IMRT in the 276 SCM muscles were as follows: grade 0, 3.3% (9/276); grade 1, 87.7% (242/276); grade 2, 9.1% (25/276); and grade 3, 0% (0/276). The 276 SCM muscles were divided into two groups: grade 0–1 atrophy, 90.9% of patients (251/276); and; grade 2–3 atrophy (moderate or severe muscle atrophy), 9.1% (25/276).

### Dosimetric analysis with respect to moderate or severe SCM atrophy

#### Significant dosimetric parameters and independent predictors of moderate or severe SCM atrophy

The median values and interquartile ranges of the dosimetric parameters for the SCMs with and without moderate or severe muscle atrophy are listed Table 2. The Wilcoxon rank-sum test showed that the dosimetric parameters Dmean, Dmax, D20, D25, D30, D35, D40, D45, D50, D55, D60, D65, D70, D75, D80, V20, V25, V30, V35, V45, V55, V60, V65, V70 and V75 were significantly associated with the development of moderate or severe SCM atrophy. On the contrary, the V40 (*P* = 0.055), V50 (*P* = 0.132) and V80 (*P* = 0.762) were not significantly associated with moderate or severe SCM atrophy (Table 2). All of the significant dosimetric parameters were included in multivariate analysis using binary logistic regression. Moderate or severe SCM atrophy was significantly related to the V65 (*β* = 0.055, *SE* = 0.013, *OR* = 1.056, 95% *CI* = [1.029–1.084], *P* < 0.001), indicating that V65 is an independent predictor of moderate or severe SCM atrophy at 3-years post IMRT in patients with NPC.

#### Dose tolerance of the SCM for moderate or severe atrophy

**Figure 1:**
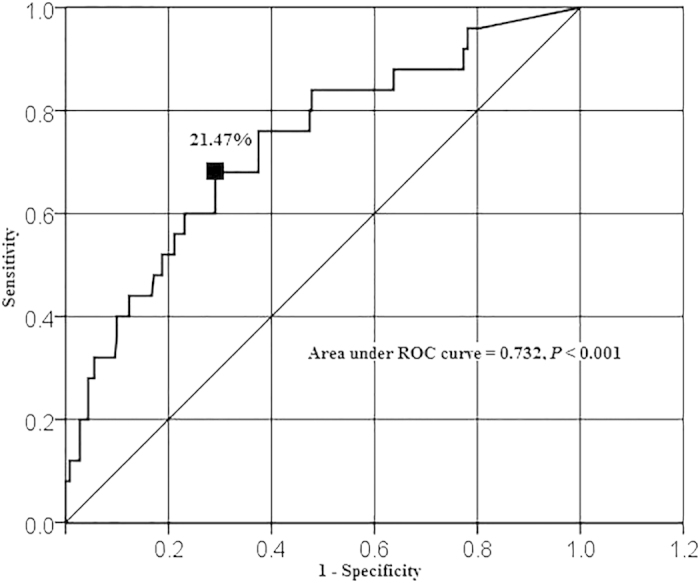
Receiver operating characteristic (ROC) curve for the V65 (percentage of the sternocleidomastoid muscle volume that received more than 65 Gy). ROC curve was generated in order to determine the dose tolerance for severe SCM atrophy. A V65 of 21.47% had a sensitivity of 0.680 and a specificity of 0.709 and was considered the dose tolerance of the SCM with respect to severe atrophy. The area under the ROC curves for a V65 of 21.47% was 0.732.

Inclusion of the V65 in ROC curve analysis was used to identify the dose tolerance cut-off point for the SCM with respect to moderate or severe atrophy. The cut-off for significant parameters was selected using the Youden index with a level of *P* < 0.05. The dose tolerance of the SCM for moderate or severe atrophy was found to be a V65 of 21.47% (sensitivity 0.680 and specificity of 0.709). The area under the ROC curves for a V65 of 21.47% was 0.732 (Fig. 1). The distribution of V65 is detailed in Table 3. A total of 186 SCMs had a V65 ≤21.47%. Of these, 178 had grade 0–1 muscle atrophy and 8 had grade 2–3 muscle atrophy. Ninety SCMs had a V65 > 21.47%. Of these, 73 had grade 0–1 muscle atrophy and 17 had grade 2–3 muscle atrophy.

### Relationship between neck muscle atrophy and neck weakness

The grades of neck weakness at 3 years post IMRT were as follows: grade 0, 47.10% (65/138); grade 1, 30.43% (42/138); grade 2, 21.01% (29/138); grade 3, 1.4% (2/138); grade 4–5, 0% (0/138). The relationship between grade of SCM atrophy and grade of neck weakness was shown in table 4. Spearman’s rank correlation test showed significant associations between the grade of neck muscle atrophy and the grade of neck muscle weakness *(R* = 0.332, *P* < 0.001).

## Discussion

Despite recent advances in radiation technology that minimize exposure of the surrounding normal tissues, many patients undergoing radiotherapy using modern techniques still experience muscle atrophy^17^,^18^,^19^,^20^. Neck muscle atrophy after radical IMRT in patients with NPC, an irreversible phenomenon that - in our experience – can develop several years after IMRT, affects muscle cell function and reduces muscle contraction force, leading to the clinical manifestation of muscle weakness. Hence, measures to reduce the occurrence of neck muscle atrophy in patients treated with IMRT are attracting more and more attention. However, the pathophysiological mechanisms leading to late-onset radiation-induced muscle atrophy are complex and not completely understood.

We explored the risk factors (age, gender, T stage, N stage, chemotherapy, the length of time after IMRT and weight change ratio) for IMRT-induced SCM atrophy in previous research. Only the N stage and the length of time after IMRT were found to be associated with IMRT-induced SCM atrophy^21^. Therefore, in the present study patients were examined 3 years after the completion of IMRT in order to exclude the effect of time on the occurrence of this late complication. Given that the N stage is related to the radiation dose delivered to the neck region, we speculated that, when the length of time after IMRT is constant, the frequency of IMRT-induced SCM atrophy will depend on the radiation dose to the neck region.

The total radiation dose received may be a significant factor in the disruption of muscle homeostasis; high dose radiation may be directly related to muscle precursor cell injury^22^,^23^. Additionally, Berthrong *et al.*^24^ postulated that radiation may indirectly affect muscles by promoting capillary and vessel injury. Therefore, we believe that the degree of muscle atrophy may be dependent on the radiation dose.

In this study, SCMs that developed grade 2–3 atrophy received a significantly higher dose of radiation than SCMs with grade 0–1 atrophy (Table 2), demonstrating that the severity of muscle atrophy at 3-years after IMRT is dependent on the radiation dose for the SCM. To our knowledge, only one case series has previously studied the relationship between the absorbed dose and atrophy of the SCM. Popovtzer *et al.*^20^ investigated the dose-effect relationship for SCM atrophy at 3 months after chemo-irradiation in head and neck cancer; however, they showed that the muscle did not become significantly thinner if the mean dose to the SCM was < 50 Gy. In contrast, a significant reduction in thickness was observed in SCMs that received a mean dose of >50 Gy. These differences indicate that SCM atrophy develops in a dose-dependent manner and that reducing the radiation dose to the SCM may effectively reduce the occurrence of severe muscle atrophy. However, Popovtzer *et al.* did not investigate the dose-effect relationship for muscle atrophy, and the dose tolerance of the SCM in terms of moderate or severe muscle atrophy had not been defined until now.

This study ascertained the dose tolerance of the SCM for moderate or severe atrophy. Grade 2–3 SCM atrophy (SCM atrophy ratio >40%) was chosen as the study endpoint, as the risk of developing neck muscle weakness appears to increase when the degree of neck muscle atrophy is higher. Our previous and current research showed significant associations between the grade of SCM atrophy and the grade of neck weakness^21^. The current study found that 9.1% of SCMs suffered moderate or severe SCM atrophy, and 22.46% of patients had grade 2–3 neck weakness, 3-years post-IMRT. The high incidence of moderate or severe SCM atrophy and grade 2–3 neck weakness and the correlation of these two syndromes confirm the need to be alert for this complication. In univariate analysis, all dosimetric parameters except for the V40, V50 and V80 were significantly associated with the occurrence of moderate and severe SCM atrophy. Multivariate analysis indicated that the V65 was independently associated with moderate or severe SCM atrophy. ROC analysis suggested that keeping the V65 of the SCM below 21.47% is likely to decrease the incidence of moderate or severe SCM atrophy.

The use of V65 cut-off value to reduce the incidence of moderate or severe SCM atrophy may be clinically important. As the prescribed dose is limited to 60–68 Gy for the PTV of the nodal gross tumour volume (GTV-N) and 54 Gy for the PTV of the CTV-2 (neck nodal regions) and the SCM is adjacent to the irradiated target volume of the cervical lymph node regions, the dose to the SCM could be too high if an established dose limit is not applied for this OAR. However, IMRT has been proven to significantly spare the adjacent normal structures without compromising tumour target coverage^9^,^25^,^26^,^27^,^28^. Therefore IMRT may be capable of reducing the dose to the SCM while simultaneously delivering high doses to the tumour targets. By delivering high-dose irradiation to defined tumour targets while minimizing the dose to surrounding normal organs and tissues, including the oral cavity, the brain/brainstem, and the spinal cord, it should be possible for radiation oncologists using IMRT technology to deliver a lower dose to the SCM as an OAR and obtain a V65 below 21.47% for the SCM.

If this dose limitation to the SCM is not possible, our results still provide a theoretical basis with which to predict the degree of radiation-induced muscle atrophy likely and thereafter explore possible salvage measures to reduce the severity of the resulting muscle atrophy. To date, exercise therapy is the only treatment method considered to be effective for muscle atrophy^29^. However, there are literature reports suggesting that there are around 19 drugs which may influence muscle atrophy, and may therefore have therapeutic potential^30^.

To the best of our knowledge, this is first study to investigate the dose tolerance of the SCM for IMRT-induced moderate or severe atrophy. The limitations of this study are the same major limitations of any retrospective study. We believe that this study provides some useful data; additional practical evidence is required to assess the feasibility of applying dose limitation to the SCM during treatment planning and confirm that the dose limitations reduce the incidence of moderate or severe SCM atrophy in patients with NPC treated with IMRT.

## Conclusions

Retrospective analysis of dose-volume histograms for the bilateral SCM in 138 patients with NPC treated using IMRT demonstrated that the V65 was an independently significant variable for moderate or severe SCM atrophy. Moreover, this study provides practical guidelines that may help to optimize NPC treatment plans and reduce the occurrence of moderate or severe SCM atrophy after IMRT in patients with NPC; we suggest a V65 limit of 21.47%.

## Additional Information

**How to cite this article**: Zhang, L.-L. *et al.* Dose-volume relationships for moderate or severe neck muscle atrophy after intensity-modulated radiotherapy in patients with nasopharyngeal carcinoma. *Sci. Rep.* **5**, 18415; doi: 10.1038/srep18415 (2015).

## Figures and Tables

**Table 1:** Clinical characteristics and treatment parameters for the 138 patients with NPC.

Characteristic	No. of patients (*n* = 138)	Percentage of cohort %
*Sex*		
Male	102	73.9
Female	36	26.1
*Age (years)*		
≤50	109	79
>50	29	21
*Pathologic type*		
WHO Type 1	1	0.72
WHO Type 2	132	99.28
T stage*		
T1	12	8.7
T2	24	17.4
T3	60	43.5
T4	42	30.4
N stage*		
N0	13	9.4
N1	49	35.5
N2	60	43.5
N3	16	11.6
Clinical stage*		
I	2	1.4
II	14	10.1
III	67	48.6
IV	55	39.9
*Chemotherapy*		
No	14	10.1
Yes	124	89.9

^*^According to the 7^th^ edition of the AJCC/UICC staging system.

**Table 2:** Comparison of dosimetric parameters for SCMs with grade 0–2 atrophy and SCMs with grade 3–4 atrophy at three years after IMRT in patients with NPC.

	Grade 0–1 SCM atrophy^§^	Grade 2–3 SCM atrophy^§^	*Z*	*P-*value
	*Median (P*_25_−*P*_75_)	*Median (P*_25_−*P*_75_)		
D max	68.82 Gy (66.34 Gy–71.45 Gy)	70.97 Gy (68.54 Gy–74.05 Gy)	−2.139	0.032
D mean	56.01 Gy (40.02 Gy–60.23 Gy)	61.93 Gy (57.68 Gy–62.19 Gy)	−3.345	0.001
D20^†^	63.55 Gy (60.94 Gy–65.60 Gy)	65.95 Gy (64.05 Gy–67.88 Gy)	−3.487	<0.001
D25	62.83 Gy (60.08 Gy–64.68 Gy)	65.33 Gy (62.94 Gy–67.24 Gy)	−3.428	0.001
D30	61.99 Gy (59.25 Gy–63.84 Gy)	64.76 Gy (62.11 Gy–66.61 Gy)	−3.483	<0.001
D35	61.35 Gy (58.50 Gy–63.04 Gy)	64.10 Gy (61.34 Gy–65.68 Gy)	−3.480	0.001
D40	60.65 Gy (57.42 Gy–62.53 Gy)	63.42 Gy (60.60 Gy–64.96 Gy)	−3.506	<0.001
D45	59.80 Gy (55.79 Gy–61.66 Gy)	62.59 Gy (59.83 Gy–64.24 Gy)	−3.533	<0.001
D50	59.15 Gy (53.97 Gy–61.04 Gy)	61.82 Gy (59.06 Gy–63.58 Gy)	−3.682	<0.001
D55	58.42 Gy (49.85 Gy–60.45 Gy)	61.14 Gy (58.26 Gy–62.72 Gy)	−3.513	<0.001
D60	57.53 Gy (43.11 Gy–59.76 Gy)	60.35 Gy (57.36 Gy–61.95 Gy)	−3.336	0.001
D65	56.62 Gy (30.70 Gy–59.05 Gy)	59.68 Gy (56.22 Gy–60.86 Gy)	−3.191	0.001
D70	55.29 Gy (18.13 Gy–58.18 Gy)	58.82 Gy (54.17 Gy–59.96 Gy)	−2.990	0.003
D75	53.74 Gy (9.32 Gy −57.16 Gy)	57.51 Gy (51.77 Gy–58.61 Gy)	−2.796	0.005
D80	52.21 Gy (4.74 Gy −55.96 Gy)	56.17 Gy (48.93 Gy–57.75 Gy)	−2.542	0.011
V20*	97.04% (67.36–100.00%)	100.00% (100.00–100.00%)	−2.793	0.005
V25	94.42% (65.51–100.00%)	100.00% (100.00–100.00%)	−3.010	0.003
V30	93.28% (63.21–100.00%)	100.00% (99.95–100.00%)	−2.858	0.004
V35	91.98% (61.36–99.98%)	99.90% (99.34–100.00%)	−2.380	0.017
V40	90.00% (59.53–99.68%)	99.04% (92.52–99.73%)	−1.921	0.055
V45	87.87% (57.35–98.33%)	95.51% (85.31–97.98%)	−2.148	0.032
V50	83.69% (54.06–94.42%)	90.31% (73.19–95.30%)	−1.508	0.132
V55	71.21% (47.04–83.39%)	82.20% (65.14–87.75%)	−2.049	0.040
V60	44.25% (25.34–57.35%)	62.16% (42.52–67.77%)	−3.032	0.002
V65	9.55% (1.14–23.65%)	27.90% (16.06–41.16%)	−3.834	<0.001
V70	0.00% (0.00–0.95%)	3.45% (0.00–10.48%)	−3.426	0.001
V75	0.00% (0.00–0.00%)	0.00% (0.00–0.00%)	−2.148	0.032
V80	0.00% (0.00–0.00%)	0.00%(0.00–0.00%)	−0.303	0.762

Abbreviations: IMRT: Intensity-modulated radiotherapy; SCM: sternocleidomastoid muscle; Dmean: Mean dose to the sternocleidomastoid muscle; Dmax: Maximum dose to the sternocleidomastoid muscle; D20^†^ is the dose to 20% of the sternocleidomastoid muscle volume; V20* is the percentage of the sternocleidomastoid muscle volume that received more than 20 Gy; the other dosimetric parameters are reported in a similar manner; ^§^Grade 0: no atrophy; Grade 1: minimal to mild muscle atrophy, 0 < SCM atrophy ratio ≤40%; Grade 2: moderate atrophy, 40% < SCM atrophy ratio ≤70%; Grade 3: severe muscular atrophy, SCM atrophy ratio >70%.

**Table 3:** The distribution of V65 in 276 SCMs.

V65	Grade of SCM atrophy*	
	Grade 0–1	Grade 2–3
≤21.47% (n = 186)	178 (95.70%)	8 (4.3%)
>21.47% (n = 90)	73 (81.11%)	17 (18.89%)

Abbreviation: SCM: sternocleidomastoid muscle; V65 is the percentage of the sternocleidomastoid muscle volume that received more than 65 Gy; *Grade 0: no atrophy; Grade 1: minimal to mild muscle atrophy, 0 < SCM atrophy ratio ≤40%; Grade 2: moderate atrophy, 40% < SCM atrophy ratio ≤70%; Grade 3: severe muscular atrophy, SCM atrophy ratio >70%.

**Table 4:** Relationship between SCM atrophy and neck weakness.

Grade of SCM atrophy*	Grade of neck weakness^†^					
	Grade 0	Grade 1	Grade 2	Grade 3	Grade 4	Grade 5
Grade 0–1 (n = 251)	127 (50.60%)	81 (32.27%)	41 (16.33%)	2 (0.80%)	0 (0.00%)	0 (0.00%)
Grade 2–3 (n = 25)	3 (12.00%)	3 (12.00%)	17 (68.00%)	2 (8.00%)	0 (0.00%)	0 (0.00%)

Abbreviation: SCM: sternocleidomastoid muscle; ^†^Common Terminology Criteria for Adverse Events (CTCAE) V3.0; *Grade 0: no atrophy; Grade 1: minimal to mild muscle atrophy, 0 < SCM atrophy ratio ≤40%; Grade 2: moderate atrophy, 40% < SCM atrophy ratio ≤70%; Grade 3: severe muscular atrophy, SCM atrophy ratio >70%.
